# *DUOXA2* Variants as an Underrecognized Cause of Diffuse Goiter: A Euthyroid Adult Case Responsive to Levothyroxine

**DOI:** 10.1155/crie/9337816

**Published:** 2026-05-29

**Authors:** Hanna Deguchi-Horiuchi, Shuji Fukata, Takahiko Kogai, Yuka Ito, Takashi Akamizu

**Affiliations:** ^1^ Center for Excellence in Thyroid Care, Kuma Hospital, Kobe, Hyogo, Japan, kuma-h.or.jp; ^2^ Department of Genetic Diagnosis and Laboratory Medicine, Dokkyo Medical University, Mibu, Tochigi, Japan, dokkyomed.ac.jp

## Abstract

Hydrogen peroxide (H_2_O_2_) is an essential substrate for thyroid peroxidase (TPO) and plays a key role in iodine oxidation, organification, and coupling reactions that generate thyroid hormones. Dual oxidase 2 (DUOX2) and its maturation factor dual oxidase maturation factor 2 (DUOXA2) are critical for H_2_O_2_ production in thyroid follicular cells. Although *DUOXA2* variants typically present with congenital hypothyroidism, phenotypic variability has been reported. We present an adult woman with a diffuse thyroid goiter and normal thyroid function who was found to have compound heterozygous *DUOXA2* variants (p.Tyr138 ^∗^/p.Tyr246 ^∗^). Despite the patient being euthyroid, levothyroxine (LT4) administration led to a reduction in thyroid size. This case highlights that *DUOXA2* variants may be present in euthyroid adults with thyroid goiter and that LT4 therapy can lead to a reduction in thyroid volume. Long‐term follow‐up may clarify the natural history and therapeutic response in euthyroid adults with *DUOXA2* variants.

## 1. Introduction

Hydrogen peroxide (H_2_O_2_) generation is a crucial step in thyroid hormone synthesis, serving as a substrate for thyroid peroxidase (TPO), which catalyzes iodine oxidation, organification (i.e., incorporating iodine to tyrosine residues in thyroglobulin), and coupling of iodotyrosines to form thyroxine (T4) and triiodothyronine (T3) [1]. Dual oxidase 1 (DUOX1) and dual oxidase 2 (DUOX2) produce H_2_O_2_ in the thyroid, and DUOX2 expression is approximately 1.5–5 times higher than those of DUOX1 [2]. Dual oxidase maturation factor 2 (DUOXA2), a maturation factor coexpressed with DUOX2, plays an important role in the endoplasmic reticulum to the Golgi apparatus transportation, maturation, and translocation to the plasma membrane of DUOX2 [3]. Since DUOXA2 plays a pivotal role in thyroid hormone synthesis, most of the few reported cases of *DUOXA2* variants have congenital hypothyroidism [4–7].

Here, we report a case of an adult woman with normal thyroid function and diffuse goiter who carried compound heterozygous *DUOXA2* variants (p.Tyr138 ^∗^/p.Tyr246 ^∗^) and experienced a reduction in thyroid size after levothyroxine (LT4) administration.

## 2. Case Presentation

A 26‐year‐old Japanese woman with a thyroid goiter presented to our hospital for further thyroid evaluation. She was enrolled in medical school at the time of her presentation. She first noticed thyroid enlargement during adolescence, with a gradual increase in size over time. She was the first child of unrelated parents and had a negative result in TSH‐based newborn screening for congenital hypothyroidism. Her mother underwent a right subtotal thyroidectomy at age 25 for a thyroid nodule that was subsequently diagnosed as a follicular adenoma, and nodules later developed in the contralateral lobe. Her father and younger brother had no notable medical history.

**Figure 1:**
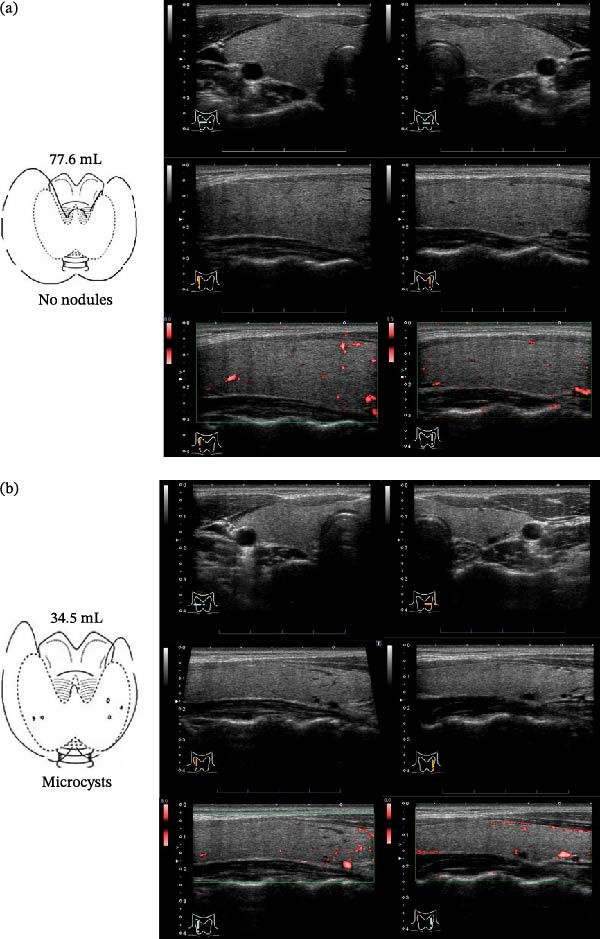
(a) Thyroid ultrasonography at the time of the patient’s initial evaluation at our hospital. The estimated thyroid volume was 77.6 mL, no nodules were detected, and no significant increase in thyroid blood flow was observed on color Doppler imaging. (b) Thyroid ultrasonography 5 years after the initiation of levothyroxine therapy. The estimated thyroid volume was 34.5 mL, microcysts were detected, and color Doppler imaging showed no significant change in thyroid blood flow compared with baseline.

The patient did not report any intolerance to cold, edema, or weight gain. She had no hearing impairment. On examination, the pulse rate was 72 beats/min. On palpation, the thyroid gland was slightly elastic, soft, and enlarged. The estimated thyroid volume measured on ultrasound was 77.6 mL, and no nodules were detected (Figure 1a). Laboratory testing using Architect (Abbott Laboratories, Illinois, USA) showed normal thyroid function: TSH 2.4171 μIU/mL (reference range: 0.3–5 μIU/mL), free T4 0.93 ng/dL (reference range: 0.7–1.6 ng/dL), free T3 2.50 pg/mL (reference range: 1.7–3.7 pg/mL). Laboratory testing using ECLusys (Roche Diagnostics, Mannheim, Germany) showed elevated thyroglobulin level of 113.40 ng/mL (reference range: 0–46.05 ng/mL). Both anti‐thyroglobulin and anti‐TPO antibodies were negative. Scintigraphy and perchlorate discharge test were not performed because of the patient’s busy schedule. LT4 therapy (50 μg/day) was initiated at age 27. At the time of reevaluation, the patient was 32 years old and was receiving a daily dose of 150 μg LT4. Laboratory analyses were conducted using ECLusys (Roche Diagnostics, Mannheim, Germany). Compared with the baseline levels before LT4 was initiated, the patient’s free T4 levels (1.73 ng/dL; reference range: 0.9–1.7 ng/dL) were slightly elevated, TSH levels were mildly reduced (1.360 μIU/mL; reference range: 0.61–4.23 μIU/mL) and free T3 levels had remained stable (2.57 pg/mL; reference range: 2.3–4 pg/mL). Both thyroglobulin levels (65.0 ng/mL; reference range: 0–46.05 ng/mL) and thyroid volume (34.5 mL) decreased over the course of treatment (Figure 1b). Additionally, no apparent change in thyroid blood flow, as assessed by color Doppler ultrasonography, was observed before and after LT4 administration (Figure 1a,b).

**Figure 2:**
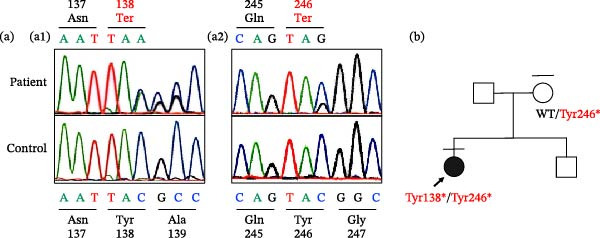
(a) Electropherograms of exon 4 and exon 5 of the *dual oxidase maturation factor 2* (*DUOXA2*) gene for the patient and a healthy control. An adenine duplication (c.413dupA) was detected in exon 4 (a1), resulting in the replacement of tyrosine (Tyr) 138 with a premature stop codon (p.Tyr138 ^∗^). In exon 5 (a2), a cytosine residue had been replaced by guanine (c.738C > G), resulting in a substitution of Tyr246 with a premature stop codon (p.Tyr246 ^∗^). (b) The black circle marked with arrow denotes the patient with compound heterozygous *DUOXA2* variants (Tyr138 ^∗^ and Tyr246 ^∗^) in the context of her familial pedigree. Her mother was found to carry Tyr246 ^∗^ heterozygous *DUOXA2* variant.

Given the patient’s family history of thyroid goiter, a dominantly inherited thyroid disorder was initially suspected. Although ultrasonography revealed a diffuse thyroid goiter, genetic testing of *DICER1* and *KEAP1* genes was performed based on her mother’s history of multinodular goiter. However, no pathogenic variants were identified. The patient had a soft goiter, but there was no clear evidence of abnormal iodine intake. Therefore, a genetic defect related to thyroid hormone synthesis was considered. Targeted next‐generation sequencing using a gene panel [8] revealed compound heterozygous variants in *DUOXA2* (p.Tyr138 ^∗^/p.Tyr246 ^∗^), which were subsequently confirmed by Sanger sequencing (Figure 2a). Subsequent genetic testing of the patient’s mother revealed a heterozygous *DUOXA2* (p.Tyr246 ^∗^) variant (Figure 2b).

## 3. Discussion

*DUOXA2* variants can result in a wide range of phenotypes, including permanent to transient or borderline congenital hypothyroidism, and may even present with normal thyroid function with or without thyroid goiter [4]. Although biallelic variants in *DUOXA2* gene are classically associated with congenital hypothyroidism, previous studies have shown that neonatal thyroid dysfunction may be mild or transient, allowing a substantial proportion of affected individuals to escape detection by TSH‐based newborn screening [5]. The negative newborn screening result in our patient is consistent with previously reported clinical patterns. Potential explanations for preserved thyroid function include residual DUOX activity, functional overlap with *DUOX1/DUOXA1*, and the contribution of maternal or placental thyroid hormone supply during the perinatal period. These compensatory mechanisms may help maintain normal thyroid hormone levels from birth through adulthood. However, a persistent mild defect in thyroid hormonogenesis may instead lead to progressive structural changes, such as goiter formation, rather than overt thyroid dysfunction, as observed in this patient.

The prevalence of *DUOXA2* variants in the general population remains difficult to estimate. Population‐based databases such as Genome Aggregation Database (gnomAD) report extremely low frequencies for specific *DUOXA2* variants, including those identified in our patient, indicating that they are rare in the general population (gnomAD v4.1.1). In contrast, disease‐specific cohort studies have demonstrated that *DUOXA2* variants are detected at measurable frequencies among patients with congenital hypothyroidism or thyroid dyshormonogenesis. For instance, *DUOXA2* variants have been reported in 12% of a United Kingdom congenital hypothyroidism cohort [5] and in 6.7% of a large Chinese congenital hypothyroidism cohort [6]. Because individuals with mild or atypical phenotypes may escape newborn screening and clinical recognition, these prevalence estimates may still underestimate the true frequency of *DUOXA2* variants. In Japan, *DUOXA2*‐related disorders have been reported mainly as sporadic case reports or small family studies, and their prevalence remains uncertain. Even in large nationwide studies of congenital hypothyroidism, the frequency of *DUOXA2* variants was not reported [9]. Importantly, the prevalence of *DUOXA2* variants in euthyroid adults with diffuse goiter has not been systematically evaluated.

Most previously reported cases of *DUOXA2* variants have been identified in neonates or children with congenital hypothyroidism, often detected by newborn screening and characterized by elevated TSH levels and impaired thyroid hormone synthesis [4–7]. In these cases, thyroid dysfunction is apparent early in life, and LT4 replacement is typically required. Previous reports also demonstrate marked phenotypic heterogeneity associated with *DUOXA2* variants. For example, Tanase‐Nakao et al. [7] described a fetus with goitrous congenital hypothyroidism and severe polyhydramnios due to compound heterozygous *DUOXA2* variants (p.Tyr138 ^∗^/p.Tyr246 ^∗^), the same variant identified in the present case, representing a profound defect in thyroid hormone synthesis manifesting during the fetal period. This report illustrates that *DUOXA2* dysfunction can lead to severe thyroid hormone synthesis defects as early as the fetal stage.

In contrast, our patient was an adult with a diffuse goiter and normal thyroid hormone levels, suggesting a mild and compensated phenotype. Adult cases with normal thyroid function are particularly rare among reported *DUOXA2*‐related disorders. The striking contrast between previously reported fetal, neonatal, or pediatric cases and our adult euthyroid case emphasizes the wide phenotypic spectrum associated with *DUOXA2* variants. Residual H_2_O_2_ generation, modifier genes, or environmental factors such as iodine intake may contribute to maintaining euthyroidism in certain individuals.

Another notable feature of our case is the decrease in thyroid volume following LT4 therapy, despite the absence of overt hypothyroidism. Although thyroid hormone replacement is crucial in congenital hypothyroidism, this observation suggests that a mild reduction in serum TSH levels may contribute to a decrease in thyroid size in some euthyroid patients with *DUOXA2* variants.

In summary, this case extends the clinical spectrum of *DUOXA2*‐related disorders to include adults with diffuse thyroid goiter and normal thyroid function. It also suggests a potential therapeutic role for LT4 in reducing the thyroid volume in such patients. To our knowledge, longitudinal data regarding changes in thyroid size in euthyroid adults with *DUOXA2* variants treated with LT4 have not been reported. Therefore, continued follow‐up of this patient may provide valuable insights into the clinical course and management of euthyroid adults with *DUOXA2* variants.

## Funding

No funding information to declare.

## Disclosure

All authors have read and approved the manuscript.

## Ethics Statement

This case report was conducted in accordance with the Declaration of Helsinki. Informed consent for publication of this case report and accompanying images was obtained from the patient.

## Conflicts of Interest

The authors declare no conflicts of interest.

## Data Availability

The data that support the findings of this study are available from the corresponding author upon reasonable request.
